# Ligustroside attenuates fibrosis-associated wound remodeling and suppresses fibrosis-related signaling in a rat excisional wound model

**DOI:** 10.1371/journal.pone.0358737

**Published:** 2026-09-25

**Authors:** Kaymin Wu, Rong Chen, Xin Liu, Youwu He

**Affiliations:** 1 Department of Plastic and Hand Surgery, Linping First People’s Hospital, Hang Zhou, Zhejiang, China; 2 Department of Basic Clinic Research, Zhejiang Province People’s Hospital, Hang Zhou, Zhejiang, China; Arizona State University, UNITED STATES OF AMERICA

## Abstract

Fibrosis-associated wound remodeling is a feature of cutaneous wound healing characterized by fibroblast activation, extracellular matrix deposition, and profibrotic signaling. Ligustroside, a major iridoid glycoside derived from *Ligustrum lucidum*, has demonstrated antifibrotic activity in several organ systems, but its effects on cutaneous wound remodeling remain unclear. In this study, a full-thickness excisional wound model was established in female Sprague–Dawley rats to investigate the effects of ligustroside on fibrosis-associated wound remodeling. Animals received ligustroside (5 or 10 mg/kg) or vehicle control. Wound formation was evaluated using the Vancouver Scar Scale, histopathological staining, and analysis of fibrosis-related markers. Ligustroside treatment significantly reduced wound severity during the remodeling phase and improved dermal architecture, as evidenced by decreased dermal thickening, reduced inflammatory infiltration, and more organized collagen fibers. Molecular analyses showed that ligustroside markedly downregulated α-smooth muscle actin and transforming growth factor-β1 expression, accompanied by showing a dose-responsive trend within the tested dose range. These findings indicate that ligustroside attenuates fibrosis-associated wound remodeling and suppresses fibrosis-related molecular responses, including TGF-β1 expression, myofibroblast activation, and extracellular matrix accumulation. Collectively, these results suggest that ligustroside may represent a promising plant-derived candidate for antifibrotic wound modulation.

## Introduction

Cutaneous wound healing involves a tightly regulated sequence of inflammatory, proliferative, and remodeling events. Dysregulated remodeling can lead to persistent fibroblast activation, excessive extracellular matrix (ECM) deposition, and fibrosis-associated wound formation [1]. Clinically, fibrosis-associated wound remodeling can lead to functional impairment, aesthetic concerns, and significant psychosocial burden, yet effective pharmacological interventions remain limited [2–6]. From an ethnopharmacological perspective, many botanical drugs traditionally used for wound care and tissue repair represent an underexplored source of bioactive metabolites with potential antifibrotic activity [7,8].

Ligustrum lucidum W.T.Aiton (Oleaceae) is a well-documented medicinal plant widely used in traditional East Asian medicine [9]. Its fruits (Ligustri Lucidi Fructus) have been traditionally prescribed for conditions associated with chronic inflammation, tissue degeneration, and impaired regeneration, and are commonly used in formulations aimed at promoting tissue repair and restoring physiological balance [10–13]. Despite its long-standing medicinal use, the molecular basis underlying its potential effects on wound healing and fibrosis-associated wound remodeling remains insufficiently characterized.

Ligustroside, also called specnuezhenide, is a major iridoid glycoside and a characteristic plant-derived metabolite of Ligustrum lucidum [14,15]. Previous pharmacological studies have demonstrated that ligustroside exhibits anti-inflammatory, antioxidant, and antifibrotic activities in experimental models of liver and lung fibrosis [16,17]. These effects have been associated with modulation of transforming growth factor-beta (TGF-β)-related signaling pathways, which play a central role in fibroblast activation, myofibroblast differentiation, and collagen synthesis [18]. However, despite the established involvement of TGF-β signaling in pathological skin remodeling, the role of ligustroside in cutaneous wound healing and fibrosis-associated wound remodeling has not been systematically investigated.

Fibrosis-associated wound remodeling is driven by persistent activation of fibroblasts and myofibroblasts, excessive deposition of collagen types I and III, and dysregulation of the TGF-β1/Smad signaling cascade. Inhibition of this pathway has been widely recognized as a promising strategy for limiting fibrosis and promoting more physiological tissue remodeling [7,19]. From an ethnopharmacological standpoint, validating whether a traditionally used botanical metabolite can modulate this key fibrotic axis in vivo provides an important bridge between empirical medicinal use and mechanism-based pharmacology.

Therefore, the present study aimed to investigate the effects of ligustroside on fibrosis-associated wound remodeling in a rat full-thickness skin wound model and to elucidate its potential mechanism of action, with particular emphasis on the TGF-β1/Smad signaling pathway. By integrating macroscopic scar assessment, histopathological analysis, and molecular evaluation of fibrotic markers, this work seeks to provide experimental evidence supporting the relevance of ligustroside as a plant-derived metabolite with therapeutic potential in scar modulation, thereby contributing to the evidence-based evaluation of traditional medicinal resources within an ethnopharmacological framework. To our knowledge, this is the first study to systematically evaluate the antifibrotic effects of ligustroside on fibrosis-associated cutaneous scar remodeling in vivo and to investigate its association with fibrosis-related molecular markers during wound healing.

## Materials and methods

### Experimental design

Female Sprague–Dawley rats were randomly assigned to three experimental groups: a model group subjected to full-thickness dorsal skin excision and receiving vehicle treatment, a ligustroside low-dose group (5 mg/kg), and a ligustroside high-dose group (10 mg/kg). Ligustroside was administered orally once daily from the day of wound modeling for 8 weeks. Scar severity was assessed longitudinally using the Vancouver Scar Scale(VSS) at weeks 1, 2, 4, and 8 [20]. Histological evaluation, immunohistochemical analysis of TGF-β1, RT-qPCR analysis of α-SMA, and western blot analysis of collagen I and III were performed at weeks 1 and 8 in all groups. This design enabled direct assessment of the antifibrotic effects of the two tested doses and their molecular correlates.antifibrotic.

### Reagents and chemicals

Ligustroside was obtained from Sichuan Cuiyirun Biotechnology Co., Ltd. (batch number: CYR-N0039230901; purity ≥ 98%). The compound was stored as a dry powder under cool, light-protected conditions. Other reagents and consumables utilized in the study include physiological saline (batch L24071313) from Sichuan Kelun Pharmaceutical Co., Ltd.; tissue fixative (batch 231014) from Nanchang Yulu Experimental Equipment Co., Ltd.; eosin staining solution (batch 20240716) from Changde Beckman Biotechnology Co., Ltd.; phosphomolybdic acid hydrate (batch C14522048) from Shanghai Macklin Biochemical Technology Co., Ltd.; acid fuchsin (batch K2123212) and Ponceau 2R (batch J2128522) both from Shanghai Aladdin Biochemical Technology Co., Ltd.; TRIzol reagent (batch 446906) from Thermo Fisher Scientific; 1st cDNA reverse transcription kit (batch TR0101) and 2 × Kappa SYBR Green I qPCR Mix (no ROX) kit (batch TR0204−5) both from Tiosbio; pre-stained protein Marker VIII (batch G2089) from Servicebio Technology Co., Ltd.; β-actin antibody (66009–1-Ig), Col I antibody (66761–1-Ig), Col III antibody (68320–1-Ig), and secondary antibody (SA00004−2) all from Proteintech Group, Inc. (Wuhan Sanying Biotechnology Co., Ltd.); α-SMA antibody (BM3092), SV ultrasensitive two-step immunohistochemistry kit (SV0002), DAB chromogenic kit (AR1027), and Anti-TGF β1 (A00019-2) all from Boster Biological Technology Co., Ltd. (Wuhan Bosterbio).

### Instruments and equipment

The instruments employed in the study encompass an analytical balance (XSR204) from Mettler-Toledo Technology (China) Co., Ltd.; an electronic balance (ME403E/02) from Mettler-Toledo Technology (China) Co., Ltd.; an ultrapure water system (SYNERGY UV) from Merck Millipore China Co., Ltd. (Shanghai); a refrigerated centrifuge (Sorvall Legend Micro21R) from Thermo Fisher Scientific (China) Co., Ltd.; a vertical automatic pressure steam sterilizer (GR110DR) from Zhiwei (Xiamen) Instrument Co., Ltd.; a microplate reader (Varioskan LUX 3020) from Thermo Fisher Scientific (China) Co., Ltd.; an upright fluorescence microscope (Axiolab 5) from Carl Zeiss Optics (China) Co., Ltd.; a paraffin embedding machine (HistoStar) from Thermo Fisher Scientific (China) Co., Ltd.; a microtome (HM 340E) from Thermo Fisher Scientific (China) Co., Ltd.; a microplate washer (WellWash) from Thermo Fisher Scientific (China) Co., Ltd.; a microplate thermostatic oscillator (MB100-2A) from Hangzhou Aosheng Instrument Co., Ltd.; a biological sample homogenizer (Bioprep-24R) from Hangzhou Aosheng Instrument Co., Ltd.; an electric heating blast drying oven (DHG-9240A) from Shanghai Yiheng Scientific Instrument Co., Ltd.; a horizontal rotator (NSP-300) from Taizhou Nuomi Medical Technology Co., Ltd.; a real-time fluorescence quantitative PCR system (qTOWER3G) from Analytik Jena AG (Germany); a PCR thermal cycler (TC-96/G/H(b)B) from Hangzhou Bioer Technology Co., Ltd.; and a microvolume spectrophotometer (Nano-300) from Hangzhou Aosheng Instrument Co., Ltd..

### Experimental animals

Specific pathogen-free (SPF) female Sprague–Dawley rats (6–8 weeks old, 180–200 g) were obtained from Zhejiang Viton Lihua Experimental Technology Co., Ltd. (license no. SCXK [Zhe] 2024−0001). Animals were acclimatized for one week prior to experimentation under controlled conditions (22 ± 2°C, 50 ± 10% humidity, 12 h light/dark cycle) with ad libitum access to food and water. All experimental procedures were conducted in accordance with institutional and national guidelines for the care and use of laboratory animals and were approved by the Animal Ethics Committee of Linping First People’s Hospital. Animals were monitored daily for general health, behavior, and signs of distress throughout the study. Body weight was recorded twice weekly. The dorsal full-thickness excisional wound model was used to investigate fibrosis-associated tissue remodeling during cutaneous wound healing. This model was not intended to reproduce specific forms of human pathological scarring. Because rodent excisional wounds differ substantially from human wound healing, particularly with respect to wound contraction and scar development, conclusions from this study were restricted to fibrosis-associated scar remodeling, collagen deposition, and related molecular responses in this experimental model.. Female rats were selected to minimize aggressive behavior and wound interference that are more commonly observed in male rodents during long-term postoperative housing. In addition, female animals generally exhibit more stable postoperative recovery and reduced variability in wound healing experiments.

### Experimental design and grouping

A total of 12 rats were randomly divided into three groups (n = 4 per group):

Model group – received distilled water by gavage;

Ligustroside 5 mg/kg group – received ligustroside at 5 mg/kg by gavage;

Ligustroside 10 mg/kg group – received ligustroside at 10 mg/kg by gavage [21].

Drug administration started on the day of modeling (designated as D1) and continued once daily for 8 consecutive weeks (56 days). Observation points were set at 1 week (D8), 2 weeks (D15), 4 weeks (D29), and 8 weeks (D57) after modeling.

The oral route of administration was selected based on previous pharmacological studies reporting systemic anti-inflammatory and antifibrotic activities of ligustroside/specnuezhenide following oral delivery in experimental fibrosis models. Oral administration also reflects the traditional clinical application pattern of Ligustrum lucidum-derived preparations in ethnopharmacological practice [16,17,21].

### Preparation of drug solutions

For the 10 mg/kg dose group, ligustroside powder was dissolved in distilled water to prepare a 1 mg/mL solution, which was freshly prepared daily. The administration volume was 0.1 mL per 10 g of body weight. The 5 mg/kg solution was prepared by two-fold dilution of the 1 mg/mL solution with distilled water to obtain a final concentration of 0.5 mg/mL.

### Establishment of the rat skin excisional wound model

All surgical procedures were performed under general anesthesia induced by intraperitoneal injection of pentobarbital sodium (40 mg/kg). Adequate depth of anesthesia was confirmed by the absence of pedal withdrawal reflex. After anesthesia, the dorsal area of each rat was shaved using an electric clipper and disinfected with povidone-iodine. Two full-thickness excisional wounds (2 cm × 2 cm each) were created symmetrically on either side of the midline, spaced 1 cm apart, and extended to the fascia layer without suturing. The wounds were left to heal by secondary intention to permit evaluation of fibrosis-associated wound remodeling during wound healing. Animals were housed individually to prevent wound interference. To minimize postoperative pain and distress, animals received appropriate supportive care, and their condition was closely monitored during recovery. Wound areas were kept clean to prevent infection.

### Clinical observation

Throughout the experiment, general health, activity, appetite, wound condition, and signs of distress were monitored at least once daily. Body weight was recorded twice weekly. Wound appearance (color, elasticity, vascularity, and thickness) was documented by photography at each observation point. In the present study, prolonged remodeling-associated fibrosis and excessive extracellular matrix deposition were used as indicators of fibrosis-associated wound remodeling.

### Humane endpoints and animal welfare considerations

Humane endpoints were predefined prior to the study to minimize animal suffering. Animals were euthanized if any of the following criteria were observed: (1) body weight loss exceeding 20% of baseline; (2) severe wound infection or necrosis; (3) persistent bleeding or non-healing wounds; (4) signs of severe distress, including lethargy, inability to access food or water, or abnormal posture; (5) self-mutilation or wound interference that could not be controlled. Animal health and behavior were monitored at least once daily throughout the study. No unexpected deaths occurred during the experimental period. All efforts were made to minimize suffering, including careful surgical technique, postoperative monitoring, and appropriate housing conditions (individual housing to prevent wound interference).

### Scar assessment

Wound healing and fibrosis-associated wound remodeling were evaluated at 1, 2, 4, and 8 weeks post-modeling using the Vancouver Scar Scale (VSS), which assesses pigmentation, vascularity, thickness, and pliability. Pigmentation was scored from 0 to 3, with 0 indicating scar color similar to adjacent normal skin, 1 representing lighter coloration, 2 mixed pigmentation, and 3 darker pigmentation. Vascularity was graded from 0 to 3, where 0 indicated normal appearance, 1 pink coloration with slightly increased blood supply, 2 red coloration with markedly increased vascularity, and 3 purple or dark red coloration with abundant blood supply. Scar thickness was scored from 0 to 3, with 0 indicating height equal to surrounding skin, 1 elevation ≤2 mm, 2 elevation >2 mm and ≤5 mm, and 3 elevation >5 mm. Pliability was evaluated on a 0–5 scale, where 0 represented normal skin, 1 soft tissue deformable under minimal resistance, 2 flexible tissue deformable under pressure, 3 firm inelastic tissue resistant to pressure, 4 banding characterized by rope-like tissue retracting upon extension, and 5 contracture reflecting permanent shortening leading to deformity. The total VSS score was calculated by summing all parameters, with higher scores indicating more severe fibrosis-associated wound remodeling. All evaluations were independently performed by two blinded observers.

### Euthanasia

At designated endpoints (week 8), animals were humanely euthanized under deep anesthesia induced by an overdose of pentobarbital sodium (≥150 mg/kg, intraperitoneal injection), followed by confirmation of death through cessation of heartbeat and respiration.

### Histopathological and immunohistochemical examination

At 1 week and 8 weeks post-treatment, scar tissues from both sides of the dorsal wound were collected. One portion was fixed in 10% neutral buffered formalin, dehydrated, embedded in paraffin, and sectioned at 4 μm thickness. Sections were stained with hematoxylin-eosin (HE) for morphological observation and Masson’s trichrome for collagen deposition assessment. For immunohistochemistry, TGF-β1 expression was detected using a two-step EnVision method (Bioss, Wuhan), with 3,3′-diaminobenzidine (DAB) as chromogen. Images were captured under 5x10 (HE&Masson) and 200x(immunohistochemistry) magnification.

### Quantitative real-time PCR (qPCR) for α-SMA mRNA expression

Total RNA was extracted from 0.2 g of frozen scar tissue using TRIzol reagent(1W & 8W). Data represent results from 4 independent animals per group (n = 4). Each tissue sample was analyzed in technical triplicate, and the mean of the three technical replicates was used as the expression value for each animal. Statistical analyses were performed on biological replicates only. RNA concentration and purity were determined with a NanoDrop spectrophotometer. cDNA was synthesized by reverse transcription using the Tiosbio 1st Strand cDNA Synthesis Kit. Quantitative PCR was performed using SYBR Green chemistry on a qTOWER3G system. The primer sequences for α-SMA were:

Forward: 5’-CAGTCGCCATCAGGAACCTC-3’.

Reverse: 5’-TTGGCCCATTCCAACCATCA-3.’

Relative gene expression was calculated using the 2^-ΔΔCt^ method, with β-actin as the internal control.

### Western blot analysis of COL-I and COL-III proteins

Approximately 100 mg of scar tissue was homogenized in RIPA lysis buffer on ice, followed by centrifugation at 12,000 rpm for 10 min at 4°C at 1 week and 8 weeks post-treatment. Protein concentrations were determined using the BCA method. Equal amounts of protein (30 μg) were separated by SDS-PAGE and transferred onto PVDF membranes. After blocking with 5% skim milk, membranes were incubated overnight at 4°C with primary antibodies against COL-I (1:2000) and COL-III (1:5000), followed by HRP-conjugated secondary antibodies (1:4000). Protein bands were visualized using enhanced chemiluminescence, and relative optical densities were analyzed using ImageJ, normalized to β-actin expression.

### Statistical analysis

All data were expressed as mean ± standard deviation (SD). Statistical analysis was performed using SPSS 25.0 software. Differences among groups were analyzed by two-way analysis of variance (ANOVA), followed by post hoc comparisons. A p-value of <0.05 was considered statistically significant; p < 0.01 and p < 0.001 were considered highly and extremely significant, respectively. All quantitative results are presented as mean ± SD, with percentage changes relative to the model group.

### Ethics approval and consent to participate

All animal experimental procedures were conducted in accordance with the ethical standards of the institutional and national guidelines for the care and use of laboratory animals. The experimental protocol was reviewed and approved by the Institutional Animal Care and Use Committee (IACUC) of Linping First People’s Hospital (LYLL202244).

## Results

### Effect of ligustroside on wound healing in the rat skin excisional wound model

**Fig 1:**
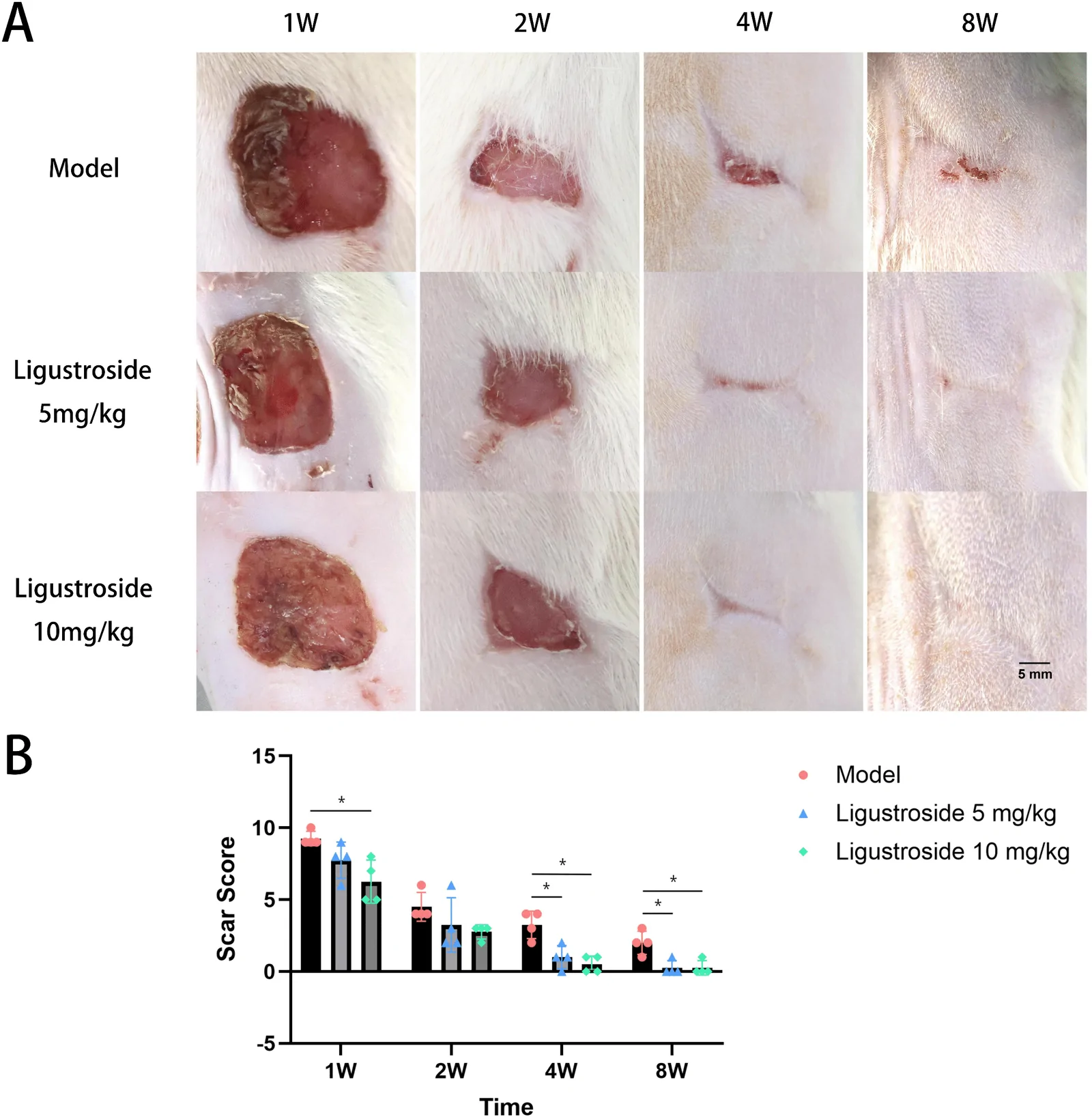
Macroscopic wound healing progression and Vancouver Scar Scale (VSS) scores following ligustroside administration. (A) Representative macroscopic images of full-thickness excisional wounds in the Model, ligustroside 5 mg/kg, and ligustroside 10 mg/kg groups at 1, 2, 4, and 8 weeks post-injury. In contrast, both ligustroside-treated groups—particularly the 10 mg/kg dose—demonstrate accelerated wound closure, smoother wound margins, and reduced pigmentation or textural irregularities over time. (B) Quantitative Vancouver Scar Scale (VSS) scores (mean ± SD; n = 4 per group) corresponding to each time point. Ligustroside treatment significantly reduced scar severity, with the 10 mg/kg group showing the greatest reduction in score. Statistical comparisons were conducted using two-way ANOVA followed by Tukey’s post hoc test. * P < 0.05, versus Model group at the same time point.

Macroscopic examination and photographic records were performed at 1, 2, 4, and 8 weeks after modeling (Fig 1A). At 1 week post-surgery, all rats exhibited stable wounds without bleeding, edema, or purulent exudation. In the ligustroside-treated groups, wound margins were partially covered by newly formed epithelium and granulation tissue. By week 2, wound contraction and epithelialization were notably enhanced in both treatment groups compared to the model group, with softer texture and improved elasticity. At week 4, complete re-epithelialization was observed in most rats receiving ligustroside, whereas unhealed regions persisted in the model group. By week 8, the 10 mg/kg ligustroside group showed almost normal skin color and texture with minimal visible scars, while the model group and 5 mg/kg group still exhibited noticeable residual scarring (Fig 1A).

In the model group, scar scores start high at 9.25 ± 0.50 in the first week and gradually decline to 4.50 ± 1.00 at 2 weeks, 3.25 ± 0.96 at 4 weeks, and 2.00 ± 0.82 at 8 weeks, reflecting a slow natural resolution of scarring without intervention. In contrast, the ligustroside 5 mg/kg group shows scores of 7.75 ± 1.26 at 1 week and 3.25 ± 1.89 at 2 weeks (without significance), but significantly lower values of 1.00 ± 0.82 at 4 weeks and 0.25 ± 0.50* at 8 weeks, demonstrating enhanced inhibition of scar formation in later stages. The higher-dose ligustroside 10 mg/kg group exhibits even earlier and more pronounced effects, with a significant reduction to 6.25 ± 1.5* at 1 week, followed by 2.75 ± 0.50 at 2 weeks (no significance), and further significant drops to 0.50 ± 0.58* at 4 weeks and 0.25 ± 0.50* at 8 weeks (Fig 1B).

The 10 mg/kg group exhibited generally greater reductions in VSS scores than the 5 mg/kg group across the evaluated time points, although the present study was not designed to establish a formal dose–response relationship. (Fig 1B). Compared with the model group, VSS scores were significantly lower in the 10 mg/kg group at week 1 (P < 0.05), and in both 5 mg/kg and 10 mg/kg groups at weeks 4 and 8 (P < 0.05), indicating that ligustroside effectively accelerated wound healing and reduced scar formation.

### Histopathological evaluation of scar tissue

**Fig 2:**
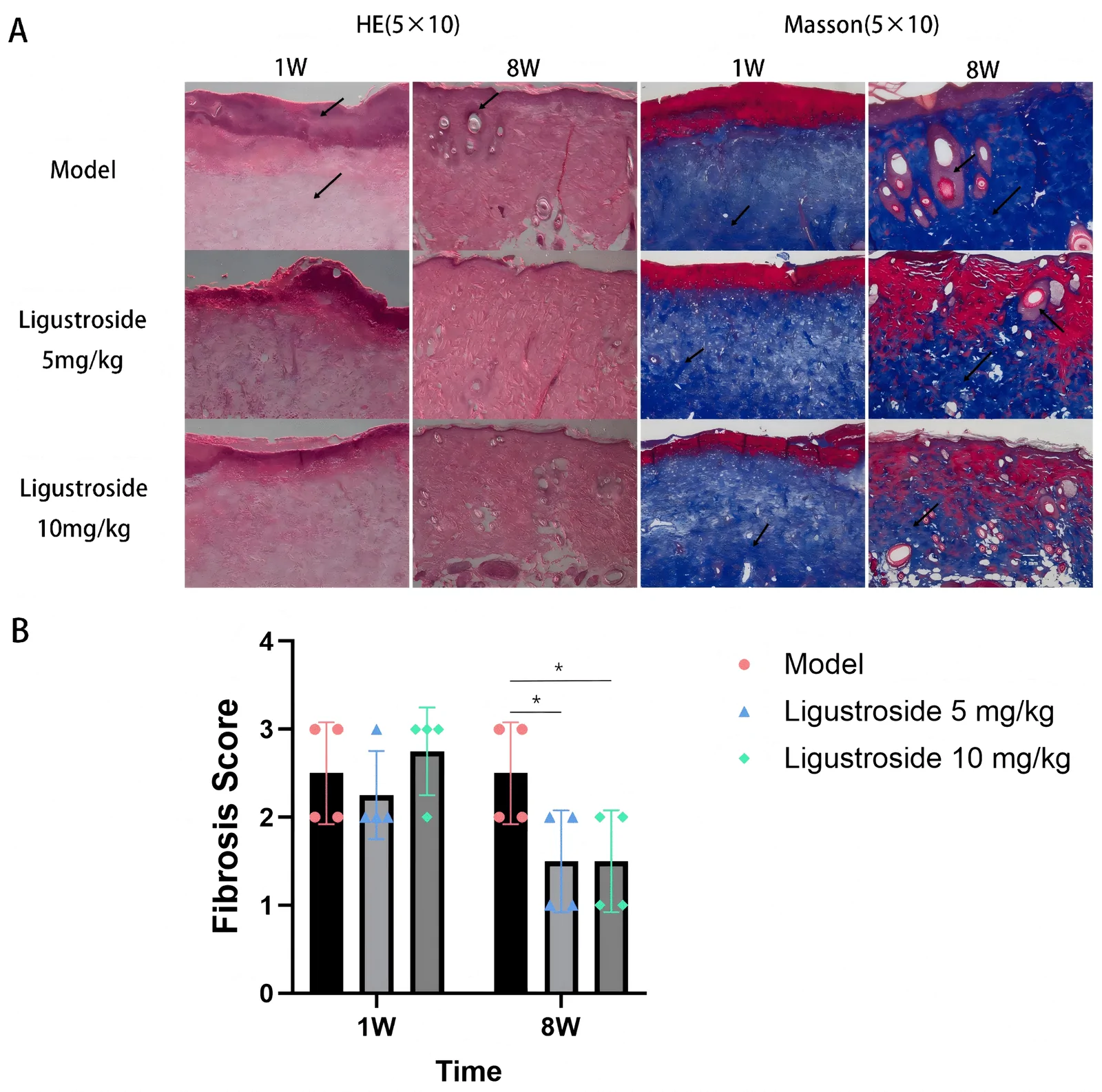
Histological analysis of wound tissue demonstrating collagen organization and fibrosis attenuation following ligustroside treatment. (A) Representative hematoxylin–eosin (H&E) stained sections from Model, ligustroside 5 mg/kg, and ligustroside 10 mg/kg groups at 1 week and 8 weeks post-injury (5x10 magnification). In the Model group, dense inflammatory infiltrates, epidermal hyperplasia, and disorganized collagen bundles are evident. At 8 weeks, the Model group shows persistent fibrotic thickening and irregular follicular structures. Ligustroside-treated groups exhibit reduced inflammatory cell infiltration at week 1 and more uniform epidermal architecture and thinner dermal collagen layers at week 8. The 10 mg/kg group demonstrates more organized tissue morphology, with improved stratification and decreased dermal compaction (black arrows). Representative Masson’s trichrome-stained sections (5x10 magnification) at the same time points. Blue-stained collagen fibers in the Model group appear densely packed, thick, and vertically oriented, consistent with fibrosis-associated wound remodeling. In contrast, ligustroside-treated tissues show reduced collagen density and more organized, horizontally aligned fiber bundles. At week 8, the 10 mg/kg group shows the most physiological collagen pattern, with decreased collagen deposition and fewer pathologically enlarged follicles (black arrows). (B) Quantitative analysis of collagen deposition (mean ± SD; n = 4 per group). Optical density (OD) values calculated from Masson’s trichrome staining demonstrate significantly lower collagen content in ligustroside groups compared with the Model group at week 8. Statistical analysis was performed using two-way ANOVA with Tukey’s post hoc test. * P < 0.05, versus Model at the same time point.

Histological changes were examined by hematoxylin-eosin (HE) and Masson’s trichrome staining at 1 and 8 weeks (Fig 2A). Arrows indicate key histopathological features, including epidermal thickening, inflammatory cell infiltration, abnormal hyperkeratosis with thickened follicular stratum corneum, and collagen fiber accumulation or disorganization(Fig 2A).

The model group maintained a consistent score of 2.50 ± 0.58 at both time points, indicating no spontaneous improvement in fibrosis. In contrast, both ligustroside-treated groups showed a significant reduction in scores by week 8 compared to the model group (P < 0.05). Specifically, the 5 mg/kg ligustroside group decreased from 2.25 ± 0.50 (1W) to 1.50 ± 0.58 (8W), while the 10 mg/kg group declined from 2.75 ± 0.50 to 1.50 ± 0.58 over the same period. Notably, although the 10 mg/kg group started with a slightly higher score at week 1, both treatment groups reached the same endpoint at week 8, demonstrating a comparable antifibrotic efficacy regardless of dose. These results highlight that ligustroside intervention, at either dose, significantly attenuated collagen deposition relative to the untreated model, supporting an inhibitory effect of ligustroside on collagen accumulation and fibrotic remodeling in this model (Fig 2B).

At week 1, scar tissue from the model group exhibited excessive dermal thickening, dense fibroblast proliferation, disorganized collagen bundles, severe inflammatory cell infiltration, and loss of dermal appendages. In contrast, ligustroside-treated rats showed reduced inflammation and better preservation of skin appendages, especially in the 10 mg/kg group(Fig 2A).

At week 8, the model group displayed markedly thickened dermis with dense, irregularly arranged collagen fibers forming whorled or wavy patterns and pronounced hyperkeratosis of hair follicles. Ligustroside-treated rats demonstrated thinner dermal layers, less collagen deposition, more organized fiber arrangement, and partial restoration of normal skin architecture(Fig 2A).

Masson’s trichrome staining confirmed a significant reduction in fibrosis scores in both the 5 mg/kg and 10 mg/kg groups compared with the model group at week 8 (P < 0.05, Fig 2B), suggesting that ligustroside mitigated collagen overaccumulation and scar tissue hypertrophy.

### Ligustroside inhibited α-SMA mRNA expression in scar tissue

**Fig 3:**
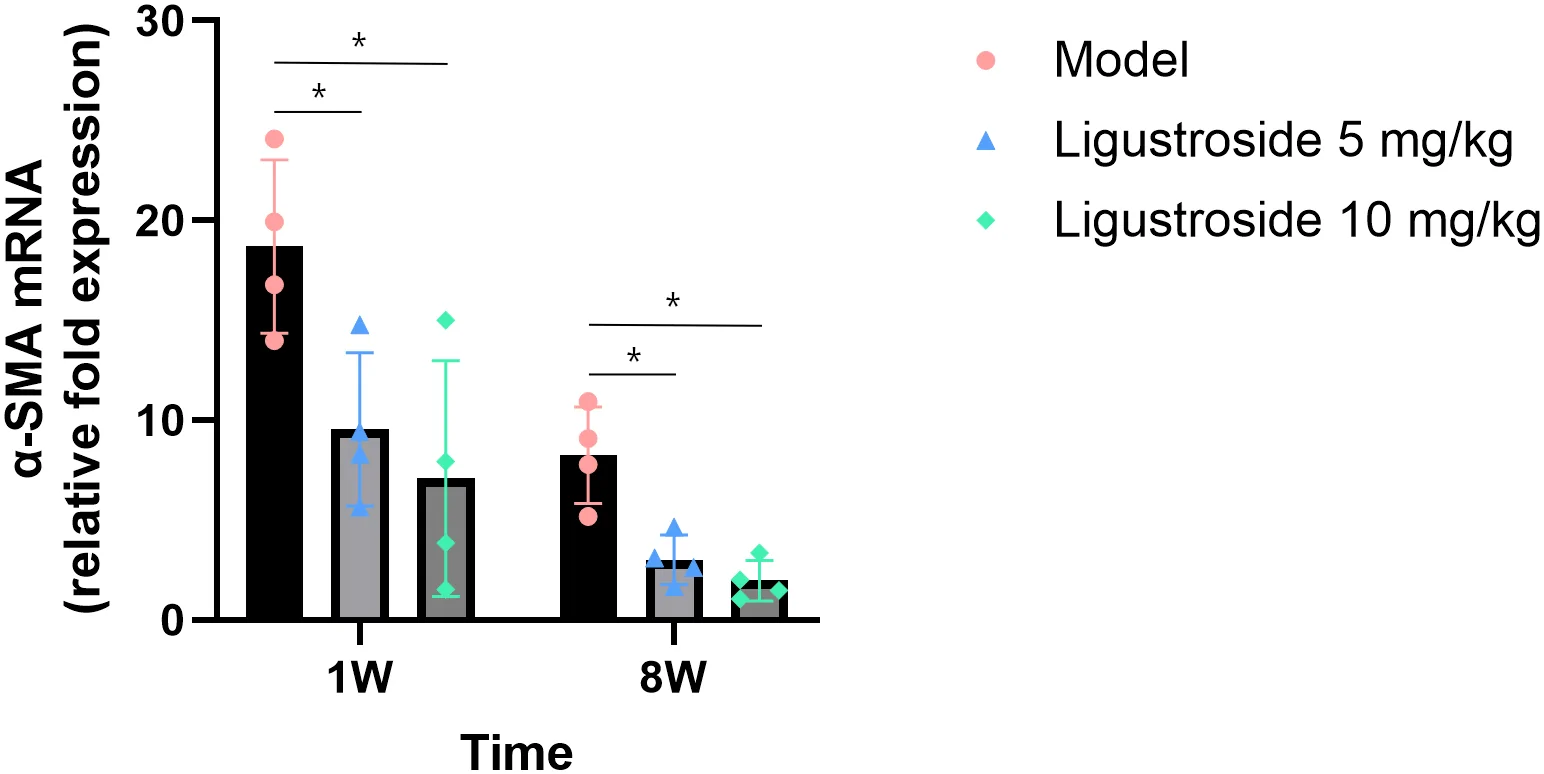
Effects of ligustroside on α-SMA mRNA expression during wound healing. Quantitative analysis of α-smooth muscle actin (α-SMA) mRNA levels in wound tissue from the Model group, ligustroside 5 mg/kg group, and ligustroside 10 mg/kg group at 1 week and 8 weeks post-injury. Expression levels were normalized to β-actin and are presented as mean ± SD (n = 4 biological replicates per group). At 1 week, both ligustroside-treated groups exhibited markedly lower α-SMA mRNA expression compared with the untreated Model group, indicating reduced early myofibroblast activation. By 8 weeks, α-SMA levels further decreased in all groups; however, both doses of ligustroside showed significantly greater suppression, with the 10 mg/kg dose demonstrating the strongest inhibitory effect. Statistical analysis was performed using two-way ANOVA followed by Tukey’s post hoc test. * P < 0.05 versus Model at the same time point.

To evaluate the effect of ligustroside on myofibroblast activation, α-smooth muscle actin (α-SMA) mRNA levels were quantified by qPCR at 1 and 8 weeks (Fig 3). At week 1, both ligustroside-treated groups showed significantly lower expression (5 mg/kg: 9.54 ± 3.83; 10 mg/kg: 7.09 ± 5.91) compared to the model group (18.70 ± 4.34), with statistical significance (P = 0.0453 and P = 0.0489, respectively). By week 8, expression levels further decreased in the treatment groups (5 mg/kg: 3.02 ± 1.23; 10 mg/kg: 1.98 ± 1.01), remaining significantly lower than the control group (8.25 ± 2.42; P = 0.0328 and P = 0.0189, respectively). Notably, while both doses of ligustroside consistently suppressed α-SMA expression relative to the model group, the 10 mg/kg group exhibited a more pronounced reduction at both time points, suggesting a dose-responsive trend inhibitory effect on this key fibrosis-associated gene. Compared with the model group, α-SMA expression was significantly reduced in both 5 mg/kg and 10 mg/kg groups at week 1 (P < 0.05) and week 8 (P < 0.05). These results indicate that ligustroside effectively inhibited myofibroblast differentiation and activity, which are key drivers of wound contraction and scar tissue formation.

### Ligustroside downregulated TGF-β1 expression in scar tissue

**Fig 4:**
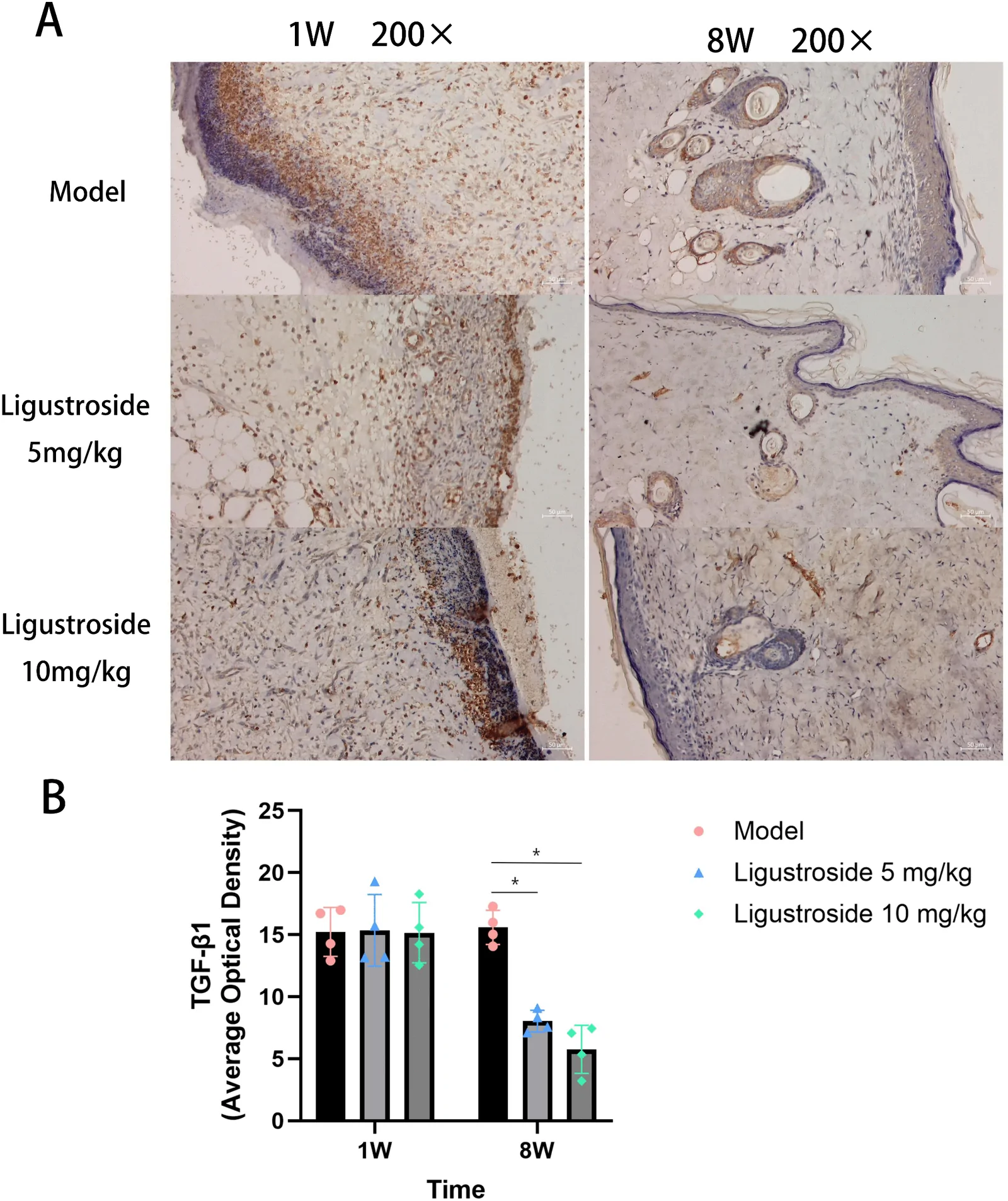
Immunohistochemical analysis of TGF-β1 expression in wound tissues following ligustroside treatment. (A) Representative immunohistochemistry (IHC) images showing TGF-β1 expression in granulation and remodeling tissue at 1 week and 8 weeks post-injury in the Model group, ligustroside 5 mg/kg group, and ligustroside 10 mg/kg group. All images were captured under identical microscopy settings (magnification ×200, scale bar embedded). Brown DAB-positive staining indicates TGF-β1-expressing cells. The Model group displayed strong TGF-β1 immunoreactivity at both time points, particularly within fibroblast-rich dermal regions. In contrast, ligustroside-treated groups demonstrated visibly reduced staining intensity, with the 10 mg/kg group showing the lowest TGF-β1 levels at 8 weeks. (B) Quantification of TGF-β1 immunoreactivity expressed as mean optical density (mean ± SD, n = 4). At 1 week, no significant differences were observed between groups (P > 0.05). At 8 weeks, both ligustroside-treated groups showed significantly reduced TGF-β1 expression compared with the Model group, consistent with attenuation of fibrosis-related signaling. Statistical analysis was performed using two-way ANOVA followed by Tukey’s post hoc test. * P < 0.05 vs. Model.

Immunohistochemical staining revealed the distribution and intensity of TGF-β1 expression in scar tissue (Fig 4A). At week 1, TGF-β levels showed no significant differences among all groups (model: 15.22 ± 2.43; 5 mg/kg: 15.35 ± 3.17, P = 0.9420; 10 mg/kg: 15.16 ± 2.43, P = 0.9732) (Fig 4B). By week 8, however, both ligustroside-treated groups exhibited markedly reduced TGF-β expression compared to the model group (15.60 ± 1.94), with the 5 mg/kg group declining to 8.03 ± 0.94 (P = 0.0005) and the 10 mg/kg group to 5.77 ± 1.94 (P = 0.0007). These results demonstrate that ligustroside significantly suppresses TGF-β protein expression in a time-dependent manner, with the higher dose showing a stronger inhibitory trend, thereby supporting its role in attenuating fibrosis-related signaling during later stages of wound healing. Brown-yellow cytoplasmic staining represented positive TGF-β1 signals. (Fig 4B). Also suggests that prolonged ligustroside administration suppresses TGF-β1 overexpression, a pivotal cytokine in extracellular matrix (ECM) accumulation and fibrotic scar development.

### Ligustroside reduced collagen type I and type III protein expression

**Fig 5:**
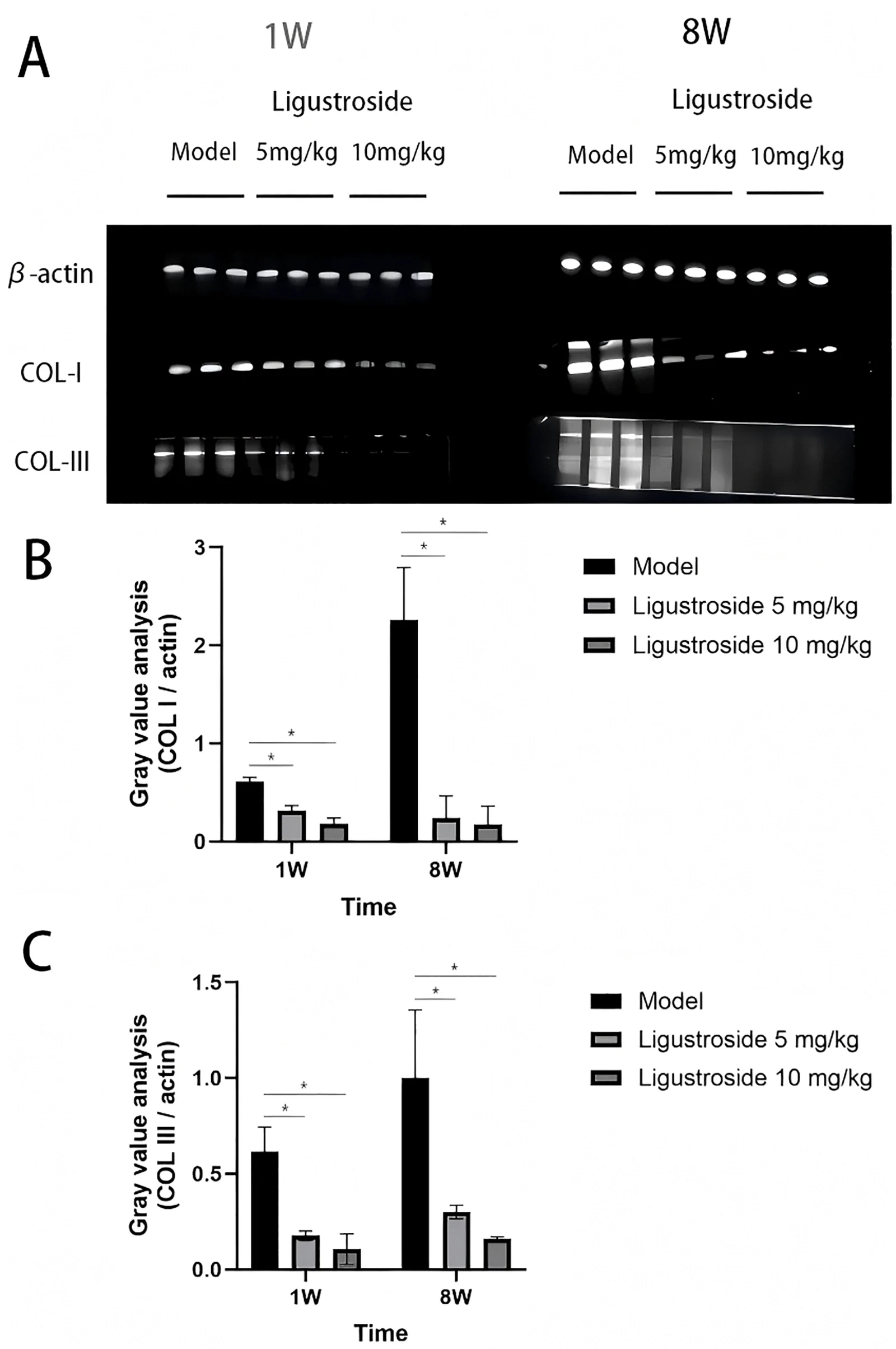
Effects of ligustroside on collagen I and collagen III expression during wound healing. (A) Representative Western blot images showing the expression of collagen type I (COL-I) and collagen type III (COL-III) in wound tissues collected at 1 week and 8 weeks post-injury from the Model group, ligustroside 5 mg/kg group, and ligustroside 10 mg/kg group. β-actin was used as the internal loading control. The Model group exhibited markedly elevated COL-I and COL-III expression at both time points, consistent with excessive extracellular matrix accumulation. In contrast, ligustroside treatment reduced collagen expression in a dose-responsive trend manner, with the 10 mg/kg group showing the lowest levels at 8 weeks. (B) Densitometric quantification of COL-I protein expression (normalized to β-actin) presented as mean ± SD (n = 4). At 1 week, both ligustroside groups showed reduced COL-I levels compared with the Model group. At 8 weeks, the Model group displayed a pronounced increase in COL-I, whereas both treatment groups demonstrated significantly suppressed expression. (C) Densitometric quantification of COL-III expression (mean ± SD, n = 4). Similar to COL-I, COL-III levels remained elevated in the Model group at 8 weeks, indicating persistent fibrosis. Ligustroside administration markedly reduced COL-III expression in a dose-responsive pattern. Statistical analysis for all densitometric data was performed using two-way ANOVA followed by Tukey’s post hoc test. * P < 0.05 vs. Model(S1-S6 Fig, S1 File).

Western blot analysis demonstrated the effects of ligustroside on COL-I and COL-III protein expression (Fig 5A–C). After 1 week of treatment, both COL-I and COL-III levels were significantly lower in the 5 mg/kg group (P < 0.01) and even more markedly reduced in the 10 mg/kg group (P < 0.001 and P < 0.01, respectively) (Fig 5B, C).

After 8 weeks, ligustroside treatment continued to suppress collagen synthesis: the 5 mg/kg group showed reduced COL-I and COL-III expression (P < 0.01 and P < 0.05), while the 10 mg/kg group exhibited more pronounced downregulation of both proteins (P < 0.01) (Fig 5B, C).

These results indicate that ligustroside significantly inhibits excessive collagen deposition, contributing to the improvement in dermal organization and reduction of scar thickness.

### Statistical summary of healing and molecular indicators

Consistent with histological and molecular analyses, the quantitative data demonstrated a dose-responsive trend improvement in wound healing quality. VSS scores, Masson fibrosis indices, α-SMA mRNA, TGF-β1 optical density, and COL-I/III expression levels all showed significant reductions compared to the model group. The 10 mg/kg ligustroside group exhibited the most pronounced therapeutic effects across all measured parameters, confirming the efficacy of ligustroside in modulating fibroblast activation, ECM deposition, and scar remodeling.

## Discussion

Cutaneous wound healing requires coordinated inflammatory, proliferative, and remodeling responses to restore tissue integrity. When remodeling becomes dysregulated, persistent fibroblast and myofibroblast activity together with excessive extracellular matrix deposition can result in fibrosis-associated changes in the repaired tissue [22–24]. In the present study, ligustroside treatment attenuated fibrosis-associated wound remodeling in a rat full-thickness excisional wound model, as demonstrated by improvements in macroscopic wound scores and dermal architecture, reduced collagen accumulation, decreased α-smooth muscle actin expression, and lower transforming growth factor-β1 expression. These findings collectively indicate that ligustroside modulates several cellular and molecular processes associated with fibrotic remodeling during cutaneous wound healing. Importantly, the present excisional wound model was used to investigate fibrosis-associated remodeling rather than to reproduce human pathological scar pathology; therefore, the findings should be interpreted within the biological scope of this experimental model.

At the macroscopic level, ligustroside treatment resulted in a time-dependent reduction in VSS scores, with generally greater effects at the higher tested dose in Vancouver Scar Scale (VSS) scores, reflecting improvements in scar color, vascularity, thickness, and pliability. Notably, significant differences emerged primarily during the later remodeling phase (weeks 4–8), rather than the early inflammatory phase. This temporal pattern suggests that ligustroside may influence fibrosis-associated tissue remodeling during later stages of wound healing, which is critical for preventing pathological scar maturation.

Histopathological analyses further support this interpretation. Compared with the untreated model group, ligustroside-treated wounds exhibited reduced dermal thickening, decreased inflammatory infiltration, and a more organized collagen fiber architecture at week 8. Masson’s trichrome staining confirmed a significant reduction in collagen accumulation, indicating attenuation of fibrotic ECM deposition rather than delayed healing. Importantly, collagen fibers in ligustroside-treated tissues exhibited a relatively more organized arrangement and reduced density compared with the untreated model group, suggesting attenuation of fibrosis-associated extracellular matrix remodeling. These findings suggest that ligustroside promotes a shift from pathological fibrosis toward physiological tissue remodeling.

At the cellular and molecular levels, ligustroside significantly suppressed α-smooth muscle actin (α-SMA) expression at both early and late time points, indicating inhibition of fibroblast-to-myofibroblast differentiation. Myofibroblasts play a central role in wound contraction and ECM production; however, their persistent activation is a hallmark of fibrosis-associated wound remodeling [25–27]. The sustained downregulation of α-SMA observed in this study suggests that ligustroside limits myofibroblast persistence, thereby preventing excessive contractile activity and collagen overproduction during scar maturation.

The concurrent reduction in TGF-β1 expression is consistent with modulation of TGF-β-associated profibrotic signaling. Because downstream Smad activation was not directly measured, the present data do not establish direct inhibition of the canonical TGF-β1/Smad pathway [28,29].. TGF-β1 is the principal profibrotic cytokine that orchestrates ECM accumulation by activating Smad2/3-mediated transcription of COL-I and COL-III [30,31]. In this study, TGF-β1 protein expression was not significantly altered during the early phase of healing, but was markedly reduced after prolonged ligustroside administration. This delayed suppression aligns with the observed effects on scar thickness and collagen deposition, supporting the notion that ligustroside primarily targets late-stage profibrotic signaling rather than acute wound responses. Consistently, downstream collagen type I and III expression was significantly decreased, indicating effective inhibition of ECM overaccumulation.

Beyond the canonical Smad pathway, it is plausible that ligustroside influences non-canonical TGF-β1 signaling cascades, including MAPK, PI3K/Akt, and JNK pathways, which regulate fibroblast proliferation, oxidative stress, and apoptosis. Given previous evidence of ligustroside analogs exhibiting antioxidant and anti-inflammatory properties, it is reasonable to speculate that ligustroside mitigates redox imbalance and cytokine overactivation within the wound microenvironment. These combined effects may contribute synergistically to reduced fibrosis and improved tissue organization.

The generally greater effects observed at 10 mg/kg than at 5 mg/kg across several fibrosis-related endpoints suggest a dose-responsive trend within the tested dose range. However, the present study evaluated only two ligustroside doses and was not designed to establish a therapeutic window. Further dose-ranging studies incorporating pharmacokinetic and systematic safety assessments will be required to define the optimal effective dose and safety margin.

From an ethnopharmacological perspective, these findings provide experimental support for the traditional use of *Ligustrum lucidum*-derived botanical drugs in conditions associated with chronic inflammation, tissue degeneration, and impaired repair. Although ligustroside is a well-characterized metabolite and has been studied in other fibrotic disease models, its role in cutaneous scar modulation has not previously been systematically evaluated [16,17]. The present study bridges this knowledge gap by demonstrating that a defined plant-derived metabolite can modulate a conserved profibrotic pathway in vivo, thereby linking traditional medicinal use with modern mechanism-based pharmacology.

Despite the promising findings, several limitations should be acknowledged. First, the sample size was relatively small (n = 4 per group), which, although sufficient for preliminary validation, may limit the statistical robustness and generalizability of the results. Future studies should employ larger cohorts and include both sexes to account for biological variability. Second, this study focused primarily on gross and molecular endpoints at fixed time points (1–8 weeks) without continuous dynamic monitoring. Real-time imaging or biomechanical assessments would provide a more comprehensive understanding of wound contraction and tissue remodeling kinetics. Third, while the results strongly suggest that the TGF-β1/Smad axis is involved, direct pathway validation (e.g., through Smad2/3 phosphorylation analysis, pathway inhibitors, or RNA interference) was not conducted. Such mechanistic experiments are necessary to definitively confirm the molecular targets of ligustroside. A further important limitation concerns the experimental model. Standard dorsal excisional wounds in rats do not reproduce the defining pathological features of human pathological scars and heal substantially through wound contraction. Accordingly, the present model was used to investigate fibrosis-associated wound remodeling rather than specific forms of human pathological scar formation, and the findings should not be interpreted as direct evidence of efficacy against human pathological scarring. Future studies employing mechanically induced or splinted scar models, large-animal models, and human scar-derived cells or tissues will be required to determine whether the antifibrotic effects observed here are relevant to human pathological scarring. In addition, conclusions regarding inflammatory cell infiltration, vascular remodeling, and detailed collagen architecture remain preliminary because specific immunohistochemical markers, including CD31, Ly6G, and F4/80, were not evaluated in the present study.

Future research should aim to deepen the mechanistic understanding of ligustroside’s antifibrotic effects and its potential integration into advanced wound-healing therapies. Key directions include:

Mechanistic elucidation: Conducting detailed molecular assays to map the interaction of ligustroside with TGF-β/Smad, MAPK, and PI3K/Akt signaling pathways.

Transcriptomic and proteomic profiling: Applying multi-omics approaches to identify global changes in gene and protein expression following ligustroside treatment.

Formulation and delivery optimization: Developing topical or nanoformulated delivery systems to enhance local drug concentration and bioavailability in wound sites.

Synergistic combination therapies: Exploring ligustroside in combination with stem cell-derived exosomes, growth factor modulators, or biomaterial scaffolds to promote scarless or regenerative healing.

Translational and clinical evaluation: Testing efficacy and safety in large-animal models and early-phase clinical studies to assess its real-world therapeutic potential.

## Conclusion

This study demonstrates that ligustroside attenuates fibrosis-associated wound remodeling in a rat full-thickness excisional wound model, as indicated by reduced wound scores, collagen accumulation, α-SMA expression, and TGF-β1 expression. The effects were generally more pronounced at 10 mg/kg than at 5 mg/kg across several endpoints. These findings support an antifibrotic effect of ligustroside during experimental wound remodeling but should not be extrapolated directly to human pathological scarring. Further studies using scar models that more closely reproduce human pathological scarring, together with direct validation of TGF-β1/Smad signaling and dose-ranging safety studies, are required to determine the translational relevance of these findings.

## Supporting information

S1 Fig WB of β-actin at 1 week. (TIF)

S2 Fig WB of β-actin at 8 week. (TIF)

S3 Fig WB of collagen type I (COL-I) at 1 week. (TIF)

S4 Fig WB of collagen type I (COL-I) at 8 week. (TIF)

S5 Fig WB of collagen type III (COL-III) at 1 week. (TIF)

S6 Fig WB of collagen type III (COL-III) at 8 week. (TIF)

S1 File Original data of WB. (XLSX)
